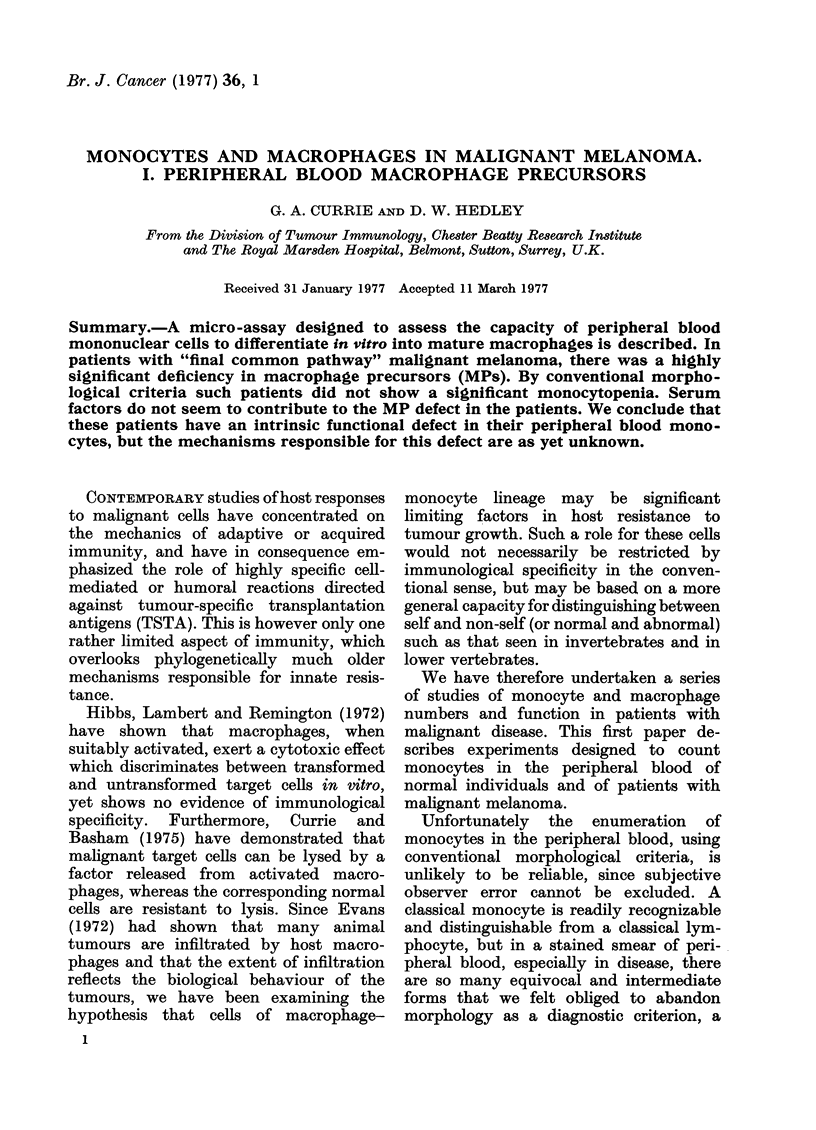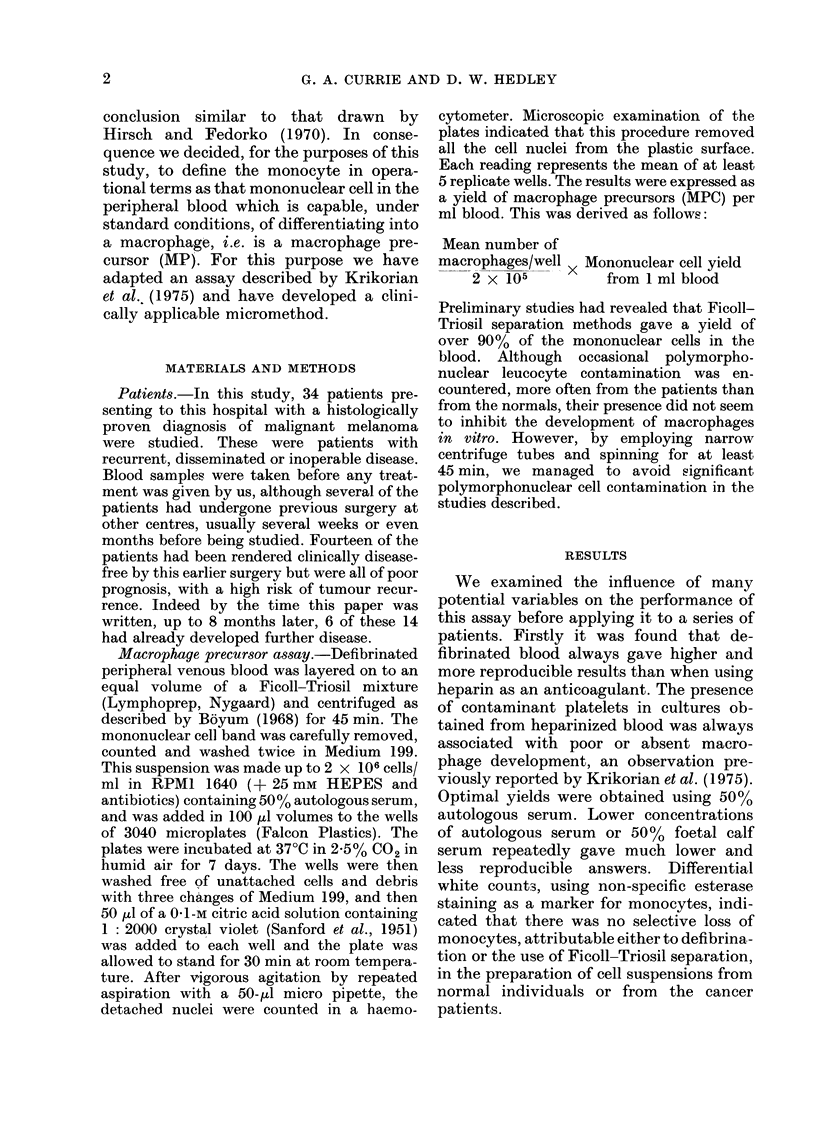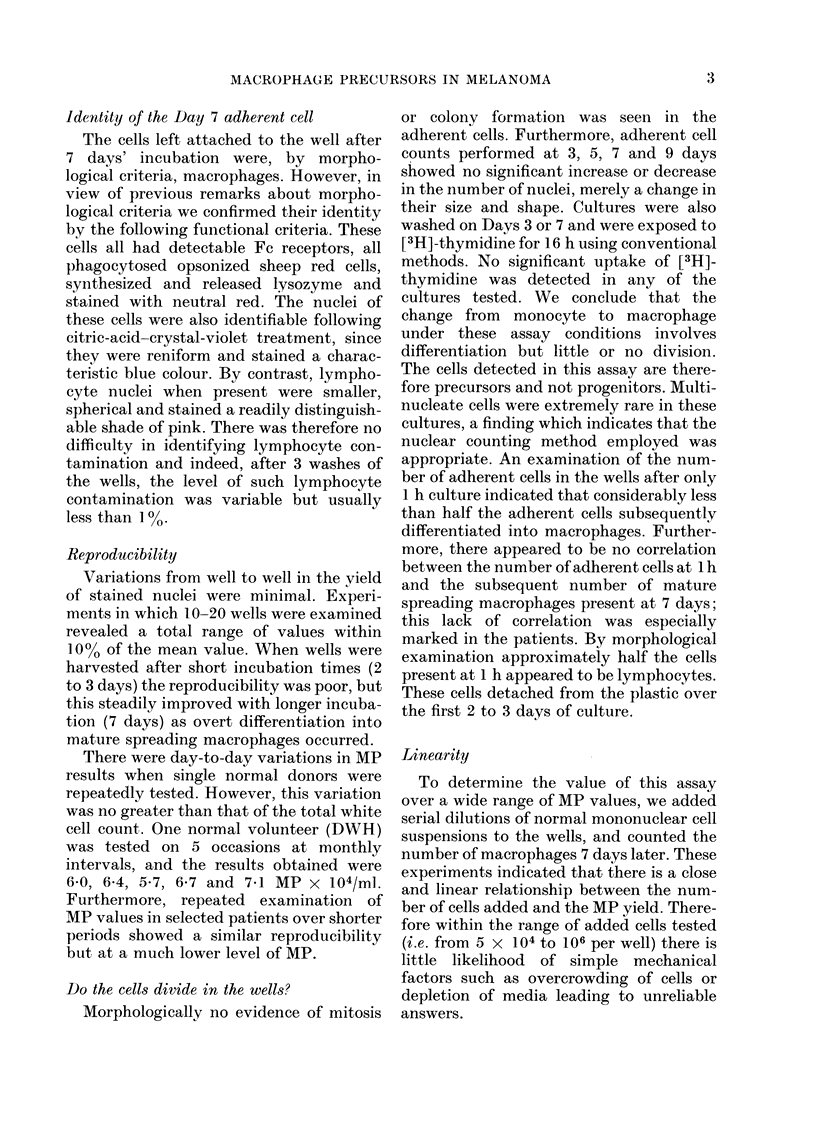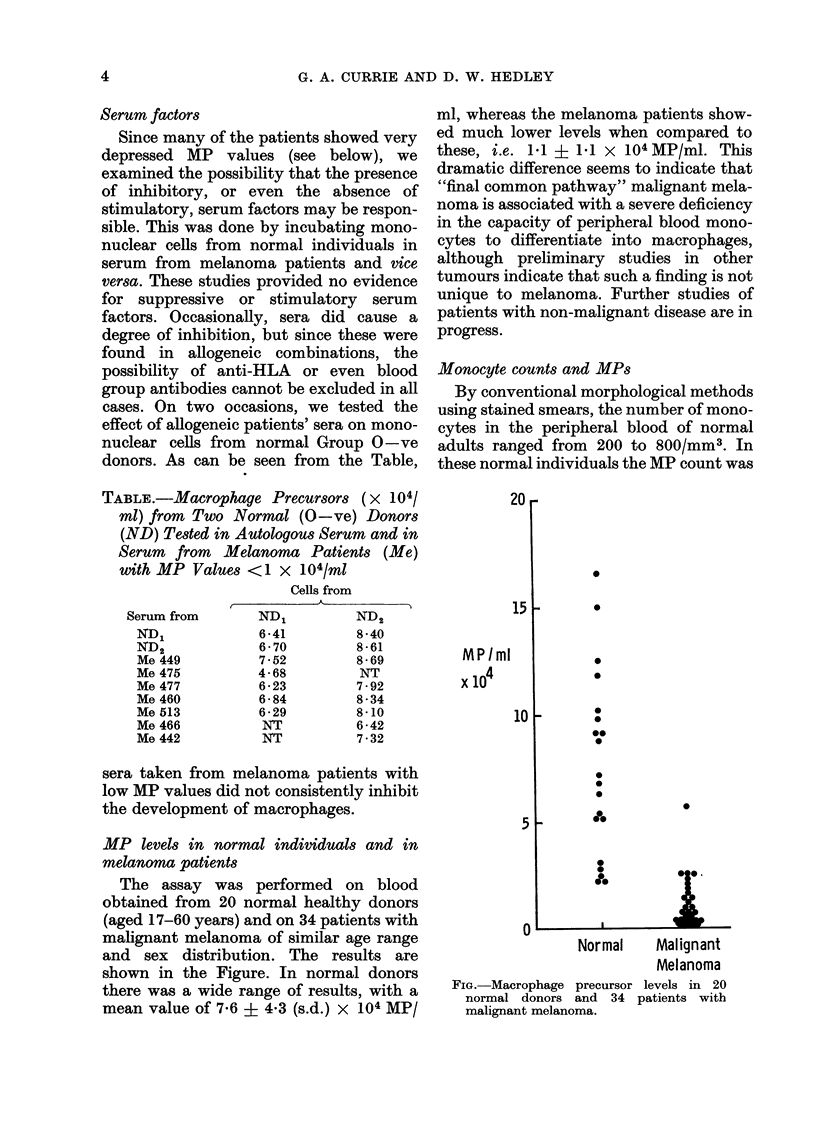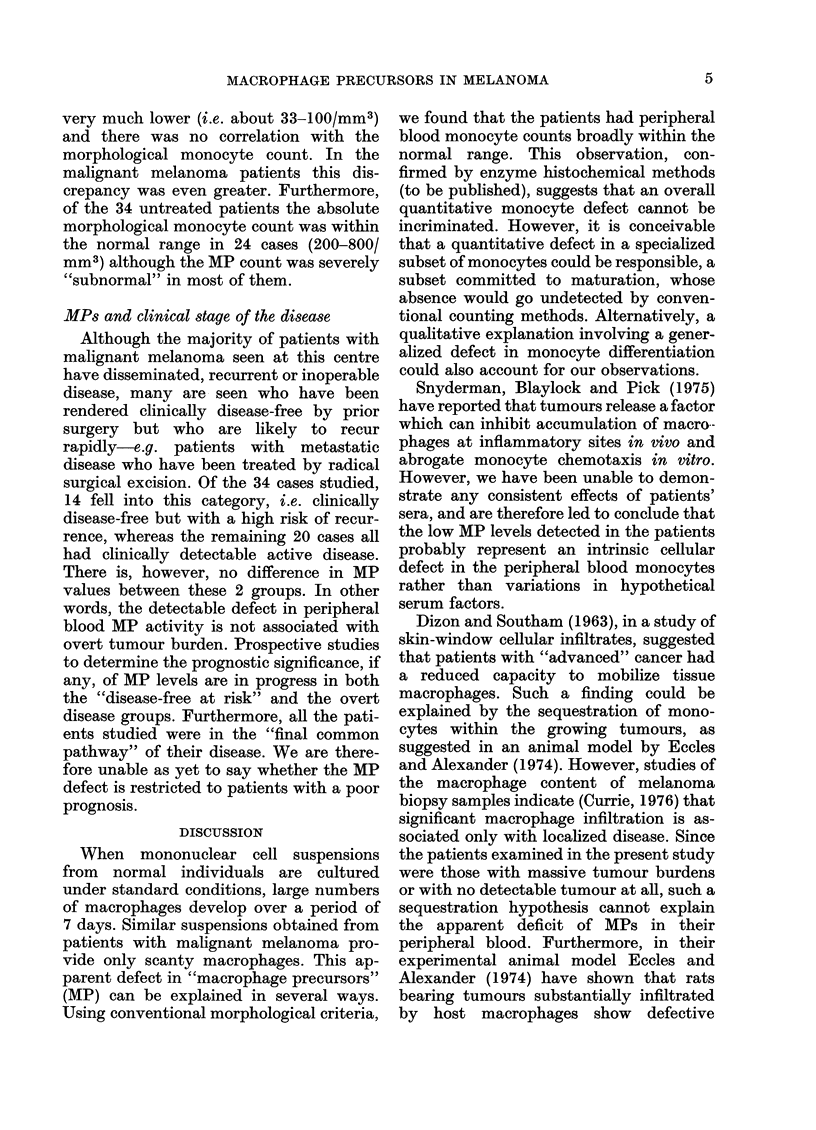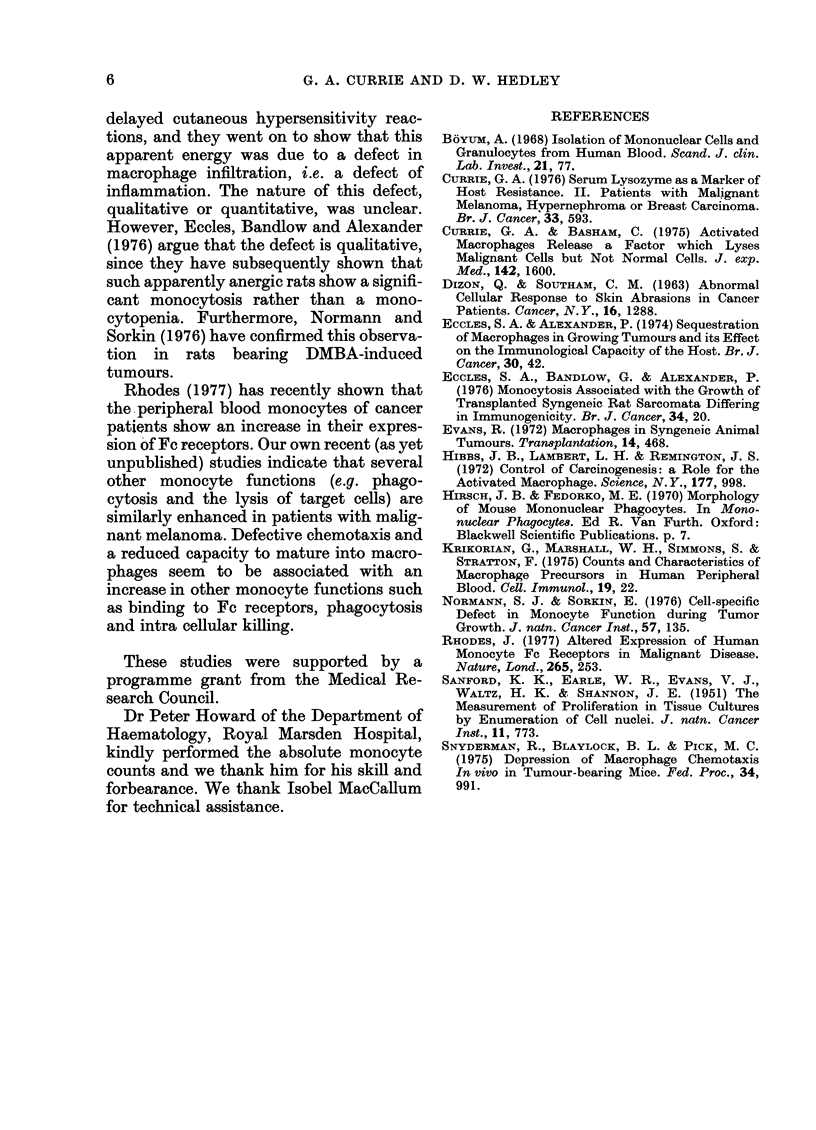# Monocytes and macrophages in malignant melanoma. I. Peripheral blood macrophage precursors.

**DOI:** 10.1038/bjc.1977.147

**Published:** 1977-07

**Authors:** G. A. Currie, D. W. Hedley

## Abstract

A micro-assay designed to assess the capacity of peripheral blood mononuclear cells to differentiate in vitro into mature macrophages is described. In patients with "final common pathway" malignant melanoma, there was a highly significant deficiency in macrophage precursors (MPs). By conventional morphological criteria such patients did not show a significant monocytopenia. Serum factors do not seem to contribute to the MP defect in the patients. We conclude that these patients have an intrinsic functional defect in their peripheral blood monocytes, but the mechanisms responsible for this defect are as yet unknown.


*Br. J. Cancer* (1977) **36**, 1

### MONOCYTES AND MACROPHAGES IN MALIGNANT MELANOMA. I. PERIPHERAL BLOOD MACROPHAGE PRECURSORS

G. A. CURRIE AND D. W. HEDLEY

*From the Division of Tumour Immunology, Chester Beatty Research Institute and The Royal Marsden Hospital, Belmont, Sutton, Surrey, U.K.*

Received 31 January 1977 Accepted 11 March 1977

**Summary.—A micro-assay designed to assess the capacity of peripheral blood mononuclear cells to differentiate *in vitro* into mature macrophages is described. In patients with "final common pathway" malignant melanoma, there was a highly significant deficiency in macrophage precursors (MPs). By conventional morphological criteria such patients did not show a significant monocytopenia. Serum factors do not seem to contribute to the MP defect in the patients. We conclude that these patients have an intrinsic functional defect in their peripheral blood monocytes, but the mechanisms responsible for this defect are as yet unknown.**

CONTEMPORARY studies of host responses to malignant cells have concentrated on the mechanics of adaptive or acquired immunity, and have in consequence emphasized the role of highly specific cell-mediated or humoral reactions directed against tumour-specific transplantation antigens (TSTA). This is however only one rather limited aspect of immunity, which overlooks phylogenetically much older mechanisms responsible for innate resistance.

Hibbs, Lambert and Remington (1972) have shown that macrophages, when suitably activated, exert a cytotoxic effect which discriminates between transformed and untransformed target cells *in vitro*, yet shows no evidence of immunological specificity. Furthermore, Currie and Basham (1975) have demonstrated that malignant target cells can be lysed by a factor released from activated macrophages, whereas the corresponding normal cells are resistant to lysis. Since Evans (1972) had shown that many animal tumours are infiltrated by host macrophages and that the extent of infiltration reflects the biological behaviour of the tumours, we have been examining the hypothesis that cells of macrophage–monocyte lineage may be significant limiting factors in host resistance to tumour growth. Such a role for these cells would not necessarily be restricted by immunological specificity in the conventional sense, but may be based on a more general capacity for distinguishing between self and non-self (or normal and abnormal) such as that seen in invertebrates and in lower vertebrates.

We have therefore undertaken a series of studies of monocyte and macrophage numbers and function in patients with malignant disease. This first paper describes experiments designed to count monocytes in the peripheral blood of normal individuals and of patients with malignant melanoma.

Unfortunately the enumeration of monocytes in the peripheral blood, using conventional morphological criteria, is unlikely to be reliable, since subjective observer error cannot be excluded. A classical monocyte is readily recognizable and distinguishable from a classical lymphocyte, but in a stained smear of peripheral blood, especially in disease, there are so many equivocal and intermediate forms that we felt obliged to abandon morphology as a diagnostic criterion, a conclusion similar to that drawn by Hirsch and Fedorko (1970). In consequence we decided, for the purposes of this study, to define the monocyte in operational terms as that mononuclear cell in the peripheral blood which is capable, under standard conditions, of differentiating into a macrophage, *i.e.* is a macrophage precursor (MP). For this purpose we have adapted an assay described by Krikorian *et al.* (1975) and have developed a clinically applicable micromethod.

### MATERIALS AND METHODS

*Patients.*—In this study, 34 patients presenting to this hospital with a histologically proven diagnosis of malignant melanoma were studied. These were patients with recurrent, disseminated or inoperable disease. Blood samples were taken before any treatment was given by us, although several of the patients had undergone previous surgery at other centres, usually several weeks or even months before being studied. Fourteen of the patients had been rendered clinically disease-free by this earlier surgery but were all of poor prognosis, with a high risk of tumour recurrence. Indeed by the time this paper was written, up to 8 months later, 6 of these 14 had already developed further disease.

*Macrophage precursor assay.*—Defibrinated peripheral venous blood was layered on to an equal volume of a Ficoll–Triosil mixture (Lymphoprep, Nygaard) and centrifuged as described by Böyum (1968) for 45 min. The mononuclear cell band was carefully removed, counted and washed twice in Medium 199. This suspension was made up to $2 \times 10^6$ cells/ml in RPMI 1640 (+ 25 mM HEPES and antibiotics) containing 50% autologous serum, and was added in 100 $\mu$l volumes to the wells of 3040 microplates (Falcon Plastics). The plates were incubated at 37°C in 2·5% $CO_2$ in humid air for 7 days. The wells were then washed free of unattached cells and debris with three changes of Medium 199, and then 50 $\mu$l of a 0·1-M citric acid solution containing 1 : 2000 crystal violet (Sanford *et al.*, 1951) was added to each well and the plate was allowed to stand for 30 min at room temperature. After vigorous agitation by repeated aspiration with a 50-$\mu$l micro pipette, the detached nuclei were counted in a haemocytometer. Microscopic examination of the plates indicated that this procedure removed all the cell nuclei from the plastic surface. Each reading represents the mean of at least 5 replicate wells. The results were expressed as a yield of macrophage precursors (MPC) per ml blood. This was derived as follows:

$$\frac{\text{Mean number of macrophages/well}}{2 \times 10^5} \times \text{Mononuclear cell yield from 1 ml blood}$$

Preliminary studies had revealed that Ficoll–Triosil separation methods gave a yield of over 90% of the mononuclear cells in the blood. Although occasional polymorphonuclear leucocyte contamination was encountered, more often from the patients than from the normals, their presence did not seem to inhibit the development of macrophages *in vitro*. However, by employing narrow centrifuge tubes and spinning for at least 45 min, we managed to avoid significant polymorphonuclear cell contamination in the studies described.

### RESULTS

We examined the influence of many potential variables on the performance of this assay before applying it to a series of patients. Firstly it was found that defibrinated blood always gave higher and more reproducible results than when using heparin as an anticoagulant. The presence of contaminant platelets in cultures obtained from heparinized blood was always associated with poor or absent macrophage development, an observation previously reported by Krikorian *et al.* (1975). Optimal yields were obtained using 50% autologous serum. Lower concentrations of autologous serum or 50% foetal calf serum repeatedly gave much lower and less reproducible answers. Differential white counts, using non-specific esterase staining as a marker for monocytes, indicated that there was no selective loss of monocytes, attributable either to defibrination or the use of Ficoll–Triosil separation, in the preparation of cell suspensions from normal individuals or from the cancer patients.

*Identity of the Day 7 adherent cell*

The cells left attached to the well after 7 days' incubation were, by morphological criteria, macrophages. However, in view of previous remarks about morphological criteria we confirmed their identity by the following functional criteria. These cells all had detectable Fc receptors, all phagocytosed opsonized sheep red cells, synthesized and released lysozyme and stained with neutral red. The nuclei of these cells were also identifiable following citric-acid–crystal-violet treatment, since they were reniform and stained a characteristic blue colour. By contrast, lymphocyte nuclei when present were smaller, spherical and stained a readily distinguishable shade of pink. There was therefore no difficulty in identifying lymphocyte contamination and indeed, after 3 washes of the wells, the level of such lymphocyte contamination was variable but usually less than 1%.

*Reproducibility*

Variations from well to well in the yield of stained nuclei were minimal. Experiments in which 10–20 wells were examined revealed a total range of values within 10% of the mean value. When wells were harvested after short incubation times (2 to 3 days) the reproducibility was poor, but this steadily improved with longer incubation (7 days) as overt differentiation into mature spreading macrophages occurred.

There were day-to-day variations in MP results when single normal donors were repeatedly tested. However, this variation was no greater than that of the total white cell count. One normal volunteer (DWH) was tested on 5 occasions at monthly intervals, and the results obtained were 6·0, 6·4, 5·7, 6·7 and 7·1 MP $\times 10^4$/ml. Furthermore, repeated examination of MP values in selected patients over shorter periods showed a similar reproducibility but at a much lower level of MP.

*Do the cells divide in the wells?*

Morphologically no evidence of mitosis or colony formation was seen in the adherent cells. Furthermore, adherent cell counts performed at 3, 5, 7 and 9 days showed no significant increase or decrease in the number of nuclei, merely a change in their size and shape. Cultures were also washed on Days 3 or 7 and were exposed to [$^3$H]-thymidine for 16 h using conventional methods. No significant uptake of [$^3$H]-thymidine was detected in any of the cultures tested. We conclude that the change from monocyte to macrophage under these assay conditions involves differentiation but little or no division. The cells detected in this assay are therefore precursors and not progenitors. Multinucleate cells were extremely rare in these cultures, a finding which indicates that the nuclear counting method employed was appropriate. An examination of the number of adherent cells in the wells after only 1 h culture indicated that considerably less than half the adherent cells subsequently differentiated into macrophages. Furthermore, there appeared to be no correlation between the number of adherent cells at 1 h and the subsequent number of mature spreading macrophages present at 7 days; this lack of correlation was especially marked in the patients. By morphological examination approximately half the cells present at 1 h appeared to be lymphocytes. These cells detached from the plastic over the first 2 to 3 days of culture.

*Linearity*

To determine the value of this assay over a wide range of MP values, we added serial dilutions of normal mononuclear cell suspensions to the wells, and counted the number of macrophages 7 days later. These experiments indicated that there is a close and linear relationship between the number of cells added and the MP yield. Therefore within the range of added cells tested (*i.e.* from $5 \times 10^4$ to $10^6$ per well) there is little likelihood of simple mechanical factors such as overcrowding of cells or depletion of media leading to unreliable answers.

*Serum factors*

Since many of the patients showed very depressed MP values (see below), we examined the possibility that the presence of inhibitory, or even the absence of stimulatory, serum factors may be responsible. This was done by incubating mononuclear cells from normal individuals in serum from melanoma patients and *vice versa*. These studies provided no evidence for suppressive or stimulatory serum factors. Occasionally, sera did cause a degree of inhibition, but since these were found in allogeneic combinations, the possibility of anti-HLA or even blood group antibodies cannot be excluded in all cases. On two occasions, we tested the effect of allogeneic patients' sera on mononuclear cells from normal Group O—ve donors. As can be seen from the Table,

TABLE.—*Macrophage Precursors* (× 10[4]/ *ml*) *from Two Normal* (O—ve) *Donors* (*ND*) *Tested in Autologous Serum and in Serum from Melanoma Patients* (*Me*) *with MP Values* <1 × 10[4]/*ml*

| Serum from | Cells from: $ND_1$ | $ND_2$ |
|---|---|---|
| $ND_1$ | 6·41 | 8·40 |
| $ND_2$ | 6·70 | 8·61 |
| Me 449 | 7·52 | 8·69 |
| Me 475 | 4·68 | NT |
| Me 477 | 6·23 | 7·92 |
| Me 460 | 6·84 | 8·34 |
| Me 513 | 6·29 | 8·10 |
| Me 466 | NT | 6·42 |
| Me 442 | NT | 7·32 |

sera taken from melanoma patients with low MP values did not consistently inhibit the development of macrophages.

*MP levels in normal individuals and in melanoma patients*

The assay was performed on blood obtained from 20 normal healthy donors (aged 17–60 years) and on 34 patients with malignant melanoma of similar age range and sex distribution. The results are shown in the Figure. In normal donors there was a wide range of results, with a mean value of 7·6 ± 4·3 (s.d.) × 10[4] MP/ml, whereas the melanoma patients showed much lower levels when compared to these, *i.e.* 1·1 ± 1·1 × 10[4] MP/ml. This dramatic difference seems to indicate that "final common pathway" malignant melanoma is associated with a severe deficiency in the capacity of peripheral blood monocytes to differentiate into macrophages, although preliminary studies in other tumours indicate that such a finding is not unique to melanoma. Further studies of patients with non-malignant disease are in progress.

*Monocyte counts and MPs*

By conventional morphological methods using stained smears, the number of monocytes in the peripheral blood of normal adults ranged from 200 to 800/mm³. In these normal individuals the MP count was

FIG.—Macrophage precursor levels in 20 normal donors and 34 patients with malignant melanoma.

very much lower (*i.e.* about 33–100/$mm^3$) and there was no correlation with the morphological monocyte count. In the malignant melanoma patients this discrepancy was even greater. Furthermore, of the 34 untreated patients the absolute morphological monocyte count was within the normal range in 24 cases (200–800/$mm^3$) although the MP count was severely "subnormal" in most of them.

*MPs and clinical stage of the disease*

Although the majority of patients with malignant melanoma seen at this centre have disseminated, recurrent or inoperable disease, many are seen who have been rendered clinically disease-free by prior surgery but who are likely to recur rapidly—*e.g.* patients with metastatic disease who have been treated by radical surgical excision. Of the 34 cases studied, 14 fell into this category, *i.e.* clinically disease-free but with a high risk of recurrence, whereas the remaining 20 cases all had clinically detectable active disease. There is, however, no difference in MP values between these 2 groups. In other words, the detectable defect in peripheral blood MP activity is not associated with overt tumour burden. Prospective studies to determine the prognostic significance, if any, of MP levels are in progress in both the "disease-free at risk" and the overt disease groups. Furthermore, all the patients studied were in the "final common pathway" of their disease. We are therefore unable as yet to say whether the MP defect is restricted to patients with a poor prognosis.

### DISCUSSION

When mononuclear cell suspensions from normal individuals are cultured under standard conditions, large numbers of macrophages develop over a period of 7 days. Similar suspensions obtained from patients with malignant melanoma provide only scanty macrophages. This apparent defect in "macrophage precursors" (MP) can be explained in several ways. Using conventional morphological criteria, we found that the patients had peripheral blood monocyte counts broadly within the normal range. This observation, confirmed by enzyme histochemical methods (to be published), suggests that an overall quantitative monocyte defect cannot be incriminated. However, it is conceivable that a quantitative defect in a specialized subset of monocytes could be responsible, a subset committed to maturation, whose absence would go undetected by conventional counting methods. Alternatively, a qualitative explanation involving a generalized defect in monocyte differentiation could also account for our observations.

Snyderman, Blaylock and Pick (1975) have reported that tumours release a factor which can inhibit accumulation of macrophages at inflammatory sites *in vivo* and abrogate monocyte chemotaxis *in vitro*. However, we have been unable to demonstrate any consistent effects of patients' sera, and are therefore led to conclude that the low MP levels detected in the patients probably represent an intrinsic cellular defect in the peripheral blood monocytes rather than variations in hypothetical serum factors.

Dizon and Southam (1963), in a study of skin-window cellular infiltrates, suggested that patients with "advanced" cancer had a reduced capacity to mobilize tissue macrophages. Such a finding could be explained by the sequestration of monocytes within the growing tumours, as suggested in an animal model by Eccles and Alexander (1974). However, studies of the macrophage content of melanoma biopsy samples indicate (Currie, 1976) that significant macrophage infiltration is associated only with localized disease. Since the patients examined in the present study were those with massive tumour burdens or with no detectable tumour at all, such a sequestration hypothesis cannot explain the apparent deficit of MPs in their peripheral blood. Furthermore, in their experimental animal model Eccles and Alexander (1974) have shown that rats bearing tumours substantially infiltrated by host macrophages show defective delayed cutaneous hypersensitivity reactions, and they went on to show that this apparent energy was due to a defect in macrophage infiltration, *i.e.* a defect of inflammation. The nature of this defect, qualitative or quantitative, was unclear. However, Eccles, Bandlow and Alexander (1976) argue that the defect is qualitative, since they have subsequently shown that such apparently anergic rats show a significant monocytosis rather than a monocytopenia. Furthermore, Normann and Sorkin (1976) have confirmed this observation in rats bearing DMBA-induced tumours.

Rhodes (1977) has recently shown that the peripheral blood monocytes of cancer patients show an increase in their expression of Fc receptors. Our own recent (as yet unpublished) studies indicate that several other monocyte functions (*e.g.* phagocytosis and the lysis of target cells) are similarly enhanced in patients with malignant melanoma. Defective chemotaxis and a reduced capacity to mature into macrophages seem to be associated with an increase in other monocyte functions such as binding to Fc receptors, phagocytosis and intra cellular killing.

These studies were supported by a programme grant from the Medical Research Council.

Dr Peter Howard of the Department of Haematology, Royal Marsden Hospital, kindly performed the absolute monocyte counts and we thank him for his skill and forbearance. We thank Isobel MacCallum for technical assistance.

REFERENCES

Böyum, A. (1968) Isolation of Mononuclear Cells and Granulocytes from Human Blood. *Scand. J. clin. Lab. Invest.*, **21**, 77.

Currie, G. A. (1976) Serum Lysozyme as a Marker of Host Resistance. II. Patients with Malignant Melanoma, Hypernephroma or Breast Carcinoma. *Br. J. Cancer*, **33**, 593.

Currie, G. A. & Basham, C. (1975) Activated Macrophages Release a Factor which Lyses Malignant Cells but Not Normal Cells. *J. exp. Med.*, **142**, 1600.

Dizon, Q. & Southam, C. M. (1963) Abnormal Cellular Response to Skin Abrasions in Cancer Patients. *Cancer, N.Y.*, **16**, 1288.

Eccles, S. A. & Alexander, P. (1974) Sequestration of Macrophages in Growing Tumours and its Effect on the Immunological Capacity of the Host. *Br. J. Cancer*, **30**, 42.

Eccles, S. A., Bandlow, G. & Alexander, P. (1976) Monocytosis Associated with the Growth of Transplanted Syngeneic Rat Sarcomata Differing in Immunogenicity. *Br. J. Cancer*, **34**, 20.

Evans, R. (1972) Macrophages in Syngeneic Animal Tumours. *Transplantation*, **14**, 468.

Hibbs, J. B., Lambert, L. H. & Remington, J. S. (1972) Control of Carcinogenesis: a Role for the Activated Macrophage. *Science, N.Y.*, **177**, 998.

Hirsch, J. B. & Fedorko, M. E. (1970) Morphology of Mouse Mononuclear Phagocytes. In *Mononuclear Phagocytes*. Ed R. Van Furth. Oxford: Blackwell Scientific Publications. p. 7.

Krikorian, G., Marshall, W. H., Simmons, S. & Stratton, F. (1975) Counts and Characteristics of Macrophage Precursors in Human Peripheral Blood. *Cell. Immunol.*, **19**, 22.

Normann, S. J. & Sorkin, E. (1976) Cell-specific Defect in Monocyte Function during Tumor Growth. *J. natn. Cancer Inst.*, **57**, 135.

Rhodes, J. (1977) Altered Expression of Human Monocyte Fc Receptors in Malignant Disease. *Nature, Lond.*, **265**, 253.

Sanford, K. K., Earle, W. R., Evans, V. J., Waltz, H. K. & Shannon, J. E. (1951) The Measurement of Proliferation in Tissue Cultures by Enumeration of Cell nuclei. *J. natn. Cancer Inst.*, **11**, 773.

Snyderman, R., Blaylock, B. L. & Pick, M. C. (1975) Depression of Macrophage Chemotaxis *In vivo* in Tumour-bearing Mice. *Fed. Proc.*, **34**, 991.